# Principal component-based regression models for predicting body weight from morphometric traits in Hararghe Highland sheep, Ethiopia

**DOI:** 10.1016/j.vas.2026.100918

**Published:** 2026-10-29

**Authors:** Mohammed Yousuf, Kefelegn Kebede, Yesihak Yusuf, Gemeda Duguma, Solomon Abegaz

**Affiliations:** a Dambi Dollo University, Department of Animal and Range Sciences, Dambi Dollo, Ethiopia; b Haramaya University, School of Animal and Range Sciences, Haramaya, Ethiopia; c Wollega University, Department of Animal Sciences, Nekemte, Ethiopia; d International Livestock Research Institutes , Addis Ababa, Ethiopia

**Keywords:** Hararghe Highland ewes, Body weight prediction, Morphometric traits, Principal component analysis, Ethiopia

## Abstract

In smallholder farming systems across the Ethiopian highlands, weighing scales are rarely available, and farmers rely on visual assessment to estimate sheep body weight (BW) for management. This study evaluated multivariate approaches for predicting BW from morphometric traits in Hararghe Highland ewes, Ethiopia. A cross-sectional survey was conducted on 392 ewes from three districts, recording BW and 16 measurements. Data were analysed using Pearson correlations, multicollinearity diagnostics, principal component analysis (PCA), and multiple linear regression (MLR), with LASSO and Elastic Net evaluated using 10-fold cross-validation in SAS 9.4. BW showed the strongest correlation with chest girth (r = 0.94), followed by rump height (r = 0.92), body length (r = 0.90), tail length (r = 0.90), and height at withers (r = 0.87; p < 0.05). PCA was supported by KMO = 0.93 and Bartlett’s test (p < 0.001). Two PCs (eigenvalues 11.50 and 1.57) explained 81.73% of total variance. PC1 represented body size and conformation, whereas PC2 reflected body depth and rump dimensions. The full MLR had high apparent fit (adjusted R² = 0.95) but severe multicollinearity (VIF >10), whereas PC regression removed it (VIF =1.0) at the cost of lower cross-validated accuracy (R² = 0.905 vs 0.940 for MLR). LASSO and Elastic Net (R² = 0.939) did not differ from MLR while controlling multicollinearity, and all three models outperformed PC regression and chest girth alone (P < 0.05). CG alone (R² = 0.881) offers a practical field option, whereas PC regression provides an interpretable description of body conformation but lower predictive accuracy.

## Highlights

• Chest girth was the strongest correlate of body weight in Hararghe Highland ewes.
• Two principal components explained 81.7% of morphometric variance.
• PC regression removed multicollinearity but was less accurate than full MLR.
• LASSO and Elastic Net matched MLR accuracy while controlling multicollinearity.
• Chest girth alone (R² = 0.88) is a practical field option for weight estimation.

## 1 Introduction

Ethiopia possesses one of the largest livestock populations in Africa, including approximately 42.9 million sheep, highlighting the importance of sheep production for rural livelihoods and the national economy (CSA, 2021). Sheep production is among the most widespread husbandry systems in the country, serving as a multifunctional resource central to the livelihoods, nutrition, and income of resource-poor smallholder farmers (Ayele & Urge, 2019). Indigenous sheep populations have adapted to a wide range of agroecological conditions and carry traits such as disease resistance, heat tolerance, and the ability to use poor-quality forage (Gizaw et al., 2007). Despite these adaptive advantages, indigenous breeds face increasing genetic erosion driven by uncontrolled crossbreeding, the disruption of traditional production systems, and the shift toward high-input management approaches. Reversing this trend requires breeding strategies and conservation programs grounded in reliable phenotypic and genetic data (Mirkena et al., 2012).

Characterizing livestock populations is the first step toward their sustainable use and conservation (Deribe et al., 2021). Morphometric evaluation (the systematic recording of body dimensions) has long been the standard method for describing phenotypic variation in body size and conformation (Assan, 2013). Body weight (BW) is particularly critical, enabling informed management decisions related to feeding, breeding, marketing, and healthcare (Asefa et al., 2017a). In sheep, BW is strongly and positively associated with linear body measurements, and chest girth is the most widely used single predictor because it reflects thoracic development and overall body mass (Eyduran et al., 2013; Kumar et al., 2018; Mavule et al., 2013; Yakubu, 2010). Building on this, multiple linear regression (MLR) models that incorporate several morphometric traits have been widely applied to improve BW prediction accuracy (Eyduran et al., 2013; Kumar et al., 2018).

Several studies have predicted sheep body weight from morphometric measurements using simple and multiple regression. Although these approaches produce accurate predictions, they typically rely on many strongly inter-correlated body measurements, which can compromise the stability and biological interpretability of the estimated coefficients. Prediction equations have been developed for several Ethiopian indigenous sheep populations. Melesse et al. (2013) established equations for indigenous sheep of southern Ethiopia, and Markos et al. (2023) applied stepwise regression to morphometric traits of sheep in the southwestern region. Bekele and Tadesse (2021) predicted body weight in Horro sheep of Oromia and found chest girth to be the strongest predictor, with body length and height at withers further improving accuracy. Megersa et al. (2025) compared random forest regression, support vector regression, and classification and regression trees for indigenous sheep; random forest was the most accurate, and heart girth was the most reliable predictor. Principal component analysis (PCA) addresses multicollinearity by converting correlated traits into a smaller number of orthogonal components that capture the main axes of variation (Eyduran et al., 2010). Regressing body weight (BW) on PC scores rather than raw traits removes multicollinearity, because the components are uncorrelated by definition, while retaining most of the predictive information (Kebede et al., 2022). Despite the growing use of PCA-based regression in livestock morphometrics, few studies have applied it to Ethiopian sheep (e.g., Kebede & Asaminew, 2023). Moreover, limited attention has been given to comparing dimension-reduction and penalized regression approaches under a common validation framework in Ethiopian indigenous sheep.

Previous studies in Ethiopia have mainly used simple or stepwise multiple regression, while more recent work has evaluated machine-learning algorithms. The present study addresses this gap in Hararghe Highland sheep by integrating principal component-based regression with penalized regression methods and evaluating them alongside conventional multiple regression and a practical single-trait model. To our knowledge, this is the first application of principal component regression to Hararghe Highland sheep. Whereas earlier PC-based regression work on Ethiopian sheep (Kebede & Asaminew, 2023) focused on prediction from PC factor scores, the present study additionally benchmarks PC regression against full multiple linear regression, LASSO, Elastic Net and a chest-girth-only model using identical cross-validation folds, with formal statistical comparison of predictive performance. Specifically, the study (i) identifies the major dimensions of morphometric variation using principal component analysis, (ii) evaluates whether regression on orthogonal component scores can provide a useful alternative to models based on highly correlated original traits, and (iii) compares these five models under the same 10-fold cross-validation with formal statistical testing. This approach provides both a biologically interpretable description of body conformation and an empirical assessment of the trade-off between multicollinearity control, predictive accuracy, and field practicality in Hararghe Highland ewes.

The Hararghe Highland (HH) sheep is an indigenous breed of the East Hararghe zone of eastern Ethiopia and an economically important resource for local smallholders, yet its phenotypic structure has received limited characterization using multivariate approaches. The present study therefore aimed to (i) describe variation in body weight and sixteen morphometric traits of HH ewes across three agro-ecological zones, (ii) identify the principal components underlying body conformation, and (iii) compare body weight prediction models built on the original traits, on orthogonal PC scores, and on penalized regression (LASSO and Elastic Net), using cross-validated performance and formal statistical comparison. The information generated is expected to guide selection, conservation, and management decisions for this breed.

## 2 Material and methods

### 2.1 Descriptions of the study areas

The study was conducted in the East Hararghe zone of Oromia National Regional state of Ethiopia (Fig. 1). Geographically, the zone extends between 7°31′17″ N and 9°49′16″ N latitudes and 41°10′16″ E and 42°58′51″ E longitudes. East Hararghe zone is subdivided in to three major climatic zones known to be temperate tropical highland, semi-temperate/tropical rainy midland, and lowland with the altitude ranges from 500–3405 m above sea level. This zone also receives more than 1200 mm of annual rainfall, and the mean annual temperature ranges from 10 °C to 15 °C (Yasin & Woldemariam, 2023). The major food crops in this area include sorghum, maize, wheat, barley, pulses, potato, tomato, and groundnut. Khat and vegetables are known cash crops. According to CSA (2021), East Hararghe zone has 1241,557 cattle, 314,134 sheep, 1151,462 goat, 99,949 horse, 300,707 donkey, 2275,854 poultry, and 75,029 bee hives. The prominent farming system of the study area is mixed crop-livestock production. The Hararghe Highland breed is the only sheep breed kept in the study districts; no other sheep breeds are raised in the study kebeles based on our field observation.

**Fig. 1:**
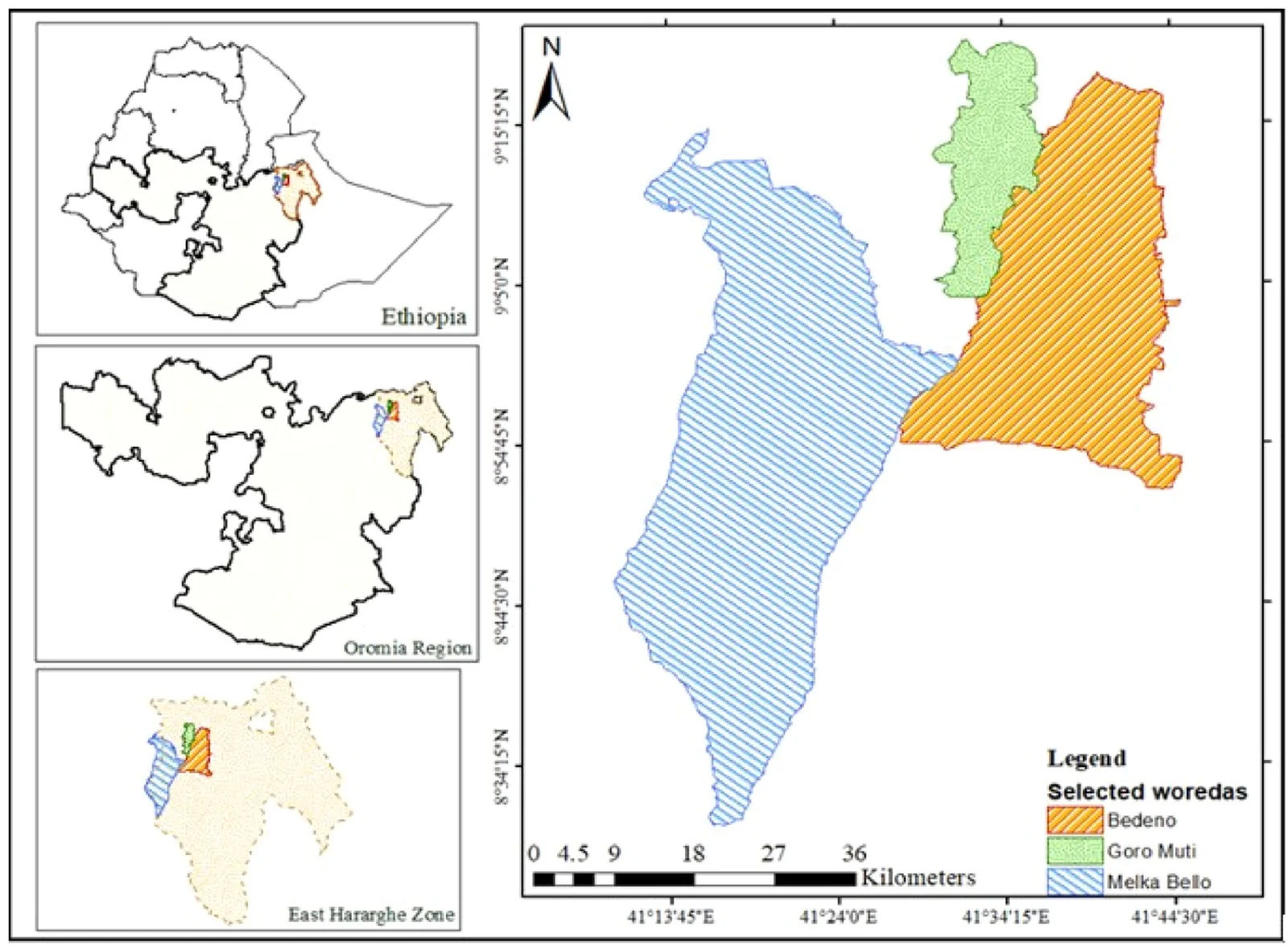
Map of the study area.

### 2.2 Study animals and sampling

A multistage purposive sampling method was utilized to gather data. First, three districts namely Melka-Balo, Bedeno, Goro-Muti in the East Hararghe zone were purposively selected to represent the lowland, midland, and highland agro-ecologies, guided by their sheep production capacity. Second, two kebeles per district were chosen based on their comparatively larger sheep populations. Lastly, the study focused on households within these kebeles that had a minimum of three mature sheep and a proven history of sheep husbandry. Accordingly, 392 HH ewes (124 from the lowland district, 130 from the midland district, and 138 from the highland district) were randomly selected from 180 households across the three districts. Animals with visible signs of disease, injury, or pregnancy were excluded from the study to minimize confounding effects on body weight and morphometric relationships. All selected animals were clinically healthy and had no visible abnormalities in body conformation. Animals aged 1 to 4 years (estimated via dentition and owner records) were grouped into five age classes (0 to 4 pairs of permanent incisors) following Wilson and Durkin (1984). All ewes were phenotypically defined as HH ewes by trained enumerators, and all flocks were reared under a traditional extensive management system. Ethical approval for the study was obtained from the School of Animal and Range Sciences Review Approval Committee, Haramaya University (Approval ID: SARS 051/24). Verbal informed consent was obtained from all livestock owners prior to the inclusion of their animals in the research. All procedures were performed in accordance with relevant institutional guidelines and professional ethical standards.

### 2.3 Morphometric trait measurements

Sheep morphometric traits were collected between October 2024 and February 2025 from smallholder farmer flocks in the three agroecological zones. Body weight (BW) and sixteen morphometric traits were measured following FAO descriptors for sheep genetic resources (FAO, 2012). The measured traits included: height-at-withers (HW), body-length (BL), chest-depth (CD), chest-girth (CG), rump-width (RW), rump-length (RL), rump-height (RH), pelvic-width (PW), shoulder- width (SW), head-width (HeW), head-length (HeL), cannon-bone-length (CBL), cannon-bone- circumference (CBC), ear-length (EL), tail-length (TL), and tail-circumference (TC).

All measurements were taken in the early morning to minimize stress-related variations, with animals in a natural standing position on level ground (Ibrahim et al., 2021). Body weight was recorded using a suspended spring balance (±0.1 kg accuracy), while linear measurements were taken using flexible measuring tapes (±0.1 cm precision) (Sun et al., 2020). The sample collection procedure involved carefully restraining each animal with the assistance of the owner to ensure safety and measurement accuracy. Each measurement was taken twice by trained personnel, and the average value was recorded to ensure accuracy. Before data collection, all observers received standardized training to ensure consistent measurements. Animals were allowed to adjust for 10–15 min before measurements to reduce stress-induced variations (Arvandi & Nugiartiningsih, 2024). Data were recorded immediately on standardized data collection sheets and later transferred to electronic format for analysis.

### 2.4 Data management and statistical analysis

All statistical analyses were performed using SAS 9.4 software (SAS Institute Inc., 2016). Following established practices in livestock morphometric traits, statistical analyses were restricted to female sheep, which formed the larger and more consistently sampled subgroup across districts (n = 392 ewes). Because body weight and most morphometric dimensions differ systematically between sexes in sheep (Bene et al., 2007), limiting the analysis to females controlled for this major source of variation, avoided confounding sex differences with trait-level relationships, and prioritized the female breeding flock which forms the foundation of genetic conservation and selection programs (Kebede & Asaminew, 2023).

#### 2.4.1 Exploratory data analysis

Body weight and morphometric traits were first explored using PROC UNIVARIATE and PROC CORR. Descriptive statistics including means, standard error, standard deviations, and coefficients of variation were computed. Furthermore, a one-way Analysis of Variance (ANOVA) using PROC GLM was conducted to evaluate the statistical variation of body weight and morphometric traits across the three agro-ecological zones/districts. Pearson correlation matrices were generated to assess relationships between variables and identify potential multicollinearity issues (Kebede et al., 2022).

### 2.5 Multicollinearity assessment

Prior to principal component analysis, multicollinearity among morphometric traits was evaluated using variance inflation factors (VIF) and tolerance values. VIF values >10 and tolerance values <0.1 were considered indicative of severe multicollinearity problems (Tahtali, 2019).

#### 2.5.1 Principal component analysis

The fitness of the dataset for factor analysis was evaluated using the Kaiser-Meyer-Olkin (KMO) measure of sampling adequacy alongside Bartlett's test of sphericity, following the recommendations of Eyduran et al. (2010). Principal component extraction was performed in SAS using the PROC FACTOR procedure with the principal-component method (METHOD=PRIN). To enhance the interpretability of the component structure, a varimax orthogonal rotation was subsequently applied.

Estimating the number of PCs: For selecting the optimal number of principal components to retain, three complementary criteria were applied: the Kaiser-Guttman eigenvalue rule, visual inspection of the scree plot, and the parallel analysis method.

Kaiser-Guttman rule: Components with eigenvalues greater than 1.0 were retained according to the Kaiser–Guttman criterion. The first component always has the largest eigenvalue, and each subsequent component explains progressively less.

Scree test: Scree plots were examined to identify the point where successive eigenvalues levelled off. This graphical display typically shows a rapid initial decline in eigenvalues, followed by a gradual levelling off. The point at which the curve forms an "elbow" or inflection marks the cut-off for component retention, indicating that additional components contribute marginally to variance explanation.

Parallel analysis: Parallel analysis was performed to verify the number of retained principal components. This approach compares the observed eigenvalues from the actual dataset with those obtained from randomly generated datasets of identical dimensions. The optimal number of components is identified at the point of convergence between the scree plot and the parallel analysis curve, ensuring that only statistically meaningful components are retained (Canaza-Cayo et al., 2021).

PC loading: Component loadings represent the correlation coefficients between the extracted principal component scores and the original measured traits. Variables exhibiting high positive loadings on PC1 are aligned with the primary axis of maximum variation in the dataset. Similarly, traits with substantial loadings on PC2 capture the second-largest source of variation, which is orthogonal to the first component.

#### 2.5.2 Body weight prediction models, cross-validation, and statistical comparison: five regression models were compared

**Model I,** chest girth only: BW = β₀ + β₁CG + ε.

**Model II**, multiple linear regression (MLR): BW = β₀ + β₁X₁ + β₂X₂ + … + β₁₆X₁₆ + ε, where Xᵢ are the 16 morphometric traits (HW, BL, CD, CG, RW, RL, RH, PW, SW, HeW, HeL, CBL, CBC, EL, TL, TC). All 16 traits were retained, without stepwise selection, to show the effect of multicollinearity before PCA transformation.

**Model III**, principal component regression (PCR): BW = β₀ + β₁PC₁ + β₂PC₂ + ε, where PC₁ and PC₂ are the scores of the two components retained according to the Kaiser–Guttman criterion, scree plot, and parallel analysis (Section 2.4.3), which together explained 81.73% of the total variance.

**Model IV**, LASSO, and **Model V**, Elastic Net: penalized regressions of BW on the 16 standardized traits, which handle multicollinearity through coefficient shrinkage and variable selection.

To address overfitting and obtain a realistic, nearly unbiased evaluation of predictive performance, internal 10-fold cross-validation was applied to all models in SAS 9.4 using macro code. All 392 complete records were randomly assigned to 10 folds of near-equal size (39 or 40 animals; random uniform numbers ranked into 10 groups; seed = 12,345). In each iteration the models were fitted on nine folds and used to predict the remaining fold, and this was repeated 10 times so that every animal was predicted once. In addition to the MLR and PC regression models, a single-trait model using chest girth (CG) only was evaluated. Two penalized regression methods, the Least Absolute Shrinkage and Selection Operator (LASSO) and Elastic Net, which handle multicollinearity through coefficient shrinkage and variable selection, were also included in the comparison. To ensure a fair comparison, identical fold assignments were used for all five models (chest-girth-only, 16-trait multiple linear regression, principal component regression, LASSO, and Elastic Net): the fold assignments were generated once and reused for every model, so that every model was trained and tested on exactly the same training and test sets within each cross-validation iteration.

To prevent information from the held-out fold from influencing any model, all model-specific steps were estimated on the training folds only in each iteration. The principal components and their regression coefficients were derived from the training data and then applied to the held-out fold. Traits were standardized using training-set statistics, and the LASSO and Elastic Net penalty parameters were tuned by an inner 5-fold cross-validation within the training data (PROC GLMSELECT, CHOOSE=CV). The number of retained components (two) was determined from the PCA of the full sample; the same number was obtained by the Kaiser criterion (eigenvalue > 1) in every training set, so this choice is unlikely to have affected the cross-validated results. Predictive accuracy on each held-out fold was evaluated using the coefficient of determination (R², calculated on the held-out animals), root mean square error (RMSE), and mean absolute error (MAE), and values are reported as mean ± SD across the 10 folds. Differences among the five models were evaluated using the fold-wise RMSE values, which were treated as paired observations because every model was evaluated on the same folds. The Friedman test (folds as blocks) was used to test for an overall difference among models, followed by pairwise Wilcoxon signed-rank tests with Holm correction for the 10 pairwise comparisons. With 10 folds, the smallest attainable two-sided exact p-value for a single Wilcoxon test is 0.002, so the procedure is conservative. The same procedure was applied to R² and MAE. Significance was declared at P < 0.05. Because training sets overlap across folds, the fold-wise estimates are not fully independent, and the resulting P-values are interpreted as indicative of relative performance rather than as exact error rates (Dietterich, 1998; Nadeau & Bengio, 2003).

## 3 Results and discussion

### 3.1 Exploratory data analysis

Table 1 presents the descriptive statistics for the body weight and morphometric traits of HH ewes. The mean body weight was 25.7 ± 0.22 kg, comparable to Asefa et al. (2017b), 26.0 kg, and Kenfo et al. (2017), 28.4 kg for indigenous Ethiopian ewes, but lower than that reported by Taye et al. (2016) (31.64 kg for Doyogena sheep), indicating breed-specific variations in body weight. Chest girth showed the highest mean (71.0 ± 0.27 cm), consistent with Gebreyowhens et al. (2016) for highland sheep. This trait is crucial because it is strongly correlated with body weight (Markos et al., 2023). The coefficient of variation values revealed substantial differences in trait variability. Height at withers had the lowest CV (5.5%), indicating that this trait varies relatively little among individual sheep. This is common in indigenous populations that have adapted to a particular environment, where skeletal height tends to be fairly uniform (Deribe et al., 2021). Conversely, the high variability observed for rump length (CV = 24.1%), cannon bone circumference (23.9%), and head width (22.2%) means that individual sheep differ considerably in these measurements. This level of variation, which is also reported in other Ethiopian sheep populations (Abera et al., 2014; Kebede & Asaminew, 2023), could be useful for selection if these traits are heritable. Body weight ranged from 15.0–33.0 kg. The observed range indicates substantial phenotypic variation in body weight within the sampled ewes. Heavier animals could be identified and selected for breeding (Areb et al., 2021). These statistics provide a comprehensive baseline for HH ewes and facilitate the use of multivariate analysis in morphometric-based body weight prediction models.

**Table 1:** Descriptive statistic results of body weight and morphometric traits of HH ewes.

No.	Trait	Label	Mean ±SE	SD	CV (%)	Min.	Max.
1	Body weight	BW	25.7 ± 0.22	4.36	17.0	15.0	33.0
2	Height at withers	HW	62.1 ± 0.17	3.42	5.5	54.0	70.0
3	Chest girth	CG	71.0 ± 0.27	5.26	7.4	57.0	80.0
4	Body length	BL	59.4 ± 0.20	4.05	6.8	53.0	71.0
5	Chest depth	CD	26.4 ± 0.22	4.38	16.5	15.0	33.0
6	Rump height	RH	62.2 ± 0.20	3.93	6.3	53.0	71.0
7	Rump length	RL	13.1 ± 0.16	3.16	24.1	5.0	22.0
8	Rump width	RW	13.3 ± 0.11	2.23	16.7	6.0	18.0
9	Pelvic width	PW	18.6 ± 0.09	1.79	9.7	13.0	22.0
10	Shoulder width	SW	14.6 ± 0.08	1.62	11.1	11.0	20.0
11	Cannon bone length	CBL	12.2 ± 0.10	1.92	15.7	7.0	17.0
12	Cannon bone circumference	CBC	7.0 ± 0.08	1.68	23.9	4.0	11.0
13	Head length	HeL	15.4 ± 0.09	1.68	11.0	12.0	19.0
14	Head width	HeW	8.1 ± 0.09	1.80	22.2	4.0	13.0
15	Ear length	EL	8.4 ± 0.09	1.75	20.7	4.0	12.0
16	Tail length	TL	18.6 ± 0.14	2.80	15.0	13.0	26.0
17	Tail circumference	TC	23.0 ± 0.14	2.85	12.4	16.0	29.0

SE= standard error of mean; SD= standard deviation; CV= coefficient of variation; Min= minimum; Max= maximum.

Table 2 presents the analysis of variance of body weight and morphometric traits of Hararghe Highland ewes across the three agroecological zones. Body weight (BW) did not differ significantly among zones, nor did HW, CD, RH, RW, CBL, CBC, EL, TL, or TC (p > 0.05). In contrast, chest girth, body length, pelvic width, shoulder width, rump length, head length, and head width varied significantly across agroecologies (p < 0.05). Lowland ewes had significantly smaller chest girth, pelvic width, and shoulder width than midland and highland ewes. Midland ewes had the narrowest rump length. Highland ewes had the shortest body length, head length, and head width.

**Table 2:** Body weight and morphometric traits of Hararghe Highland ewes across three agroecologies (mean ± SE).

Traits	Lowland	Midland	Highland
Body weight	25.66 ± 0.39^a^	25.66 ± 0.37^a^	25.70 ± 0.38^a^
Height at withers	62.11 ± 0.30^a^	61.93 ± 0.30^a^	62.13 ± 0.30^a^
Chest girth	69.90 ± 0.47^b^	71.75 ± 0.45^a^	71.36 ± 0.45^a^
Body length	59.35 ± 0.36^a^	60.23 ± 0.39^a^	58.78 ± 0.31^b^
Chest depth	26.30 ± 0.42^a^	26.90 ± 0.36^a^	26.16 ± 0.37^a^
Rump height	62.24 ± 0.33^a^	62.35 ± 0.34^a^	62.13 ± 0.37^a^
Rump length	13.95 ± 0.16^a^	12.42 ± 0.22 ^b^	13.64 ± 0.17^a^
Rump width	13.26 ± 0.29^a^	13.16 ± 0.27^a^	12.96 ± 0.27^a^
Pelvic width	18.12 ± 0.15^b^	18.99 ± 0.15^a^	18.58 ± 0.16^a^
Shoulder width	14.31 ± 0.14^b^	14.92 ± 0.16^a^	14.51 ± 0.13^a^
Cannon bone length	12.02 ± 0.16^a^	12.45 ± 0.17^a^	12.23 ± 0.17^a^
Cannon bone circumference	7.01 ± 0.14^a^	7.17 ± 0.15^a^	6.91 ± 0.15^a^
Head length	15.54 ± 0.15^a^	15.48 ± 0.15^a^	15.07 ± 0.14^b^
Head width	8.31 ± 0.15^a^	8.38 ± 0.17^a^	7.69 ± 0.14^b^
Ear length	8.40 ± 0.15^a^	8.45 ± 0.15^a^	8.48 ± 0.16^a^
Tail length	18.60 ± 0.25^a^	18.67 ± 0.25^a^	18.66 ± 0.23^a^
Tail circumference	22.95 ± 0.26^a^	22.99 ± 0.26^a^	23.14 ± 0.23^a^

Within each column, values with different superscript letters differ significantly (Holm-adjusted Wilcoxon signed-rank tests, P < 0.05); values sharing a letter do not differ significantly; RMSE = Root Mean Square Error; MAE = Mean Absolute Error; SD = Standard Deviation; CG= chest girth; MLR= multiple linear regression; PC= principal component.

Within a row, means followed by different superscript letters differ significantly at p < 0.05.

### 3.2 Pearson correlation coefficients

Table 3 presents the Pearson correlation coefficients between body weight and other morphometric traits. The correlation coefficients ranged from 0.06 (SW and RW) to 0.94 (BW and CG), demonstrating an important variation in morphometric trait relationships. Body weight showed exceptionally strong correlations with key morphometric measurements, with the highest correlation between body weight and chest girth (r = 0.94), confirming chest girth as the most reliable predictor, consistent with Markos et al. (2023), who reported similar strong correlations in Ethiopian indigenous sheep. Rump height (r = 0.92), body length (r = 0.90) and tail length (r = 0.90) also demonstrated very strong correlations, whereas height at withers showed a strong correlation (r = 0.87), supporting the use of these measurements as secondary predictors (Ibrahim et al., 2021). Moderate correlations were observed between body weight and chest depth (r = 0.72), cannon bone length (r = 0.77), and pelvic width (r = 0.75), suggesting that these traits contribute to body conformation but are less reliable as independent predictors. Among morphometric measurements, height measurements (HW and RH) showed very strong correlations (r = 0.91), indicating coordinated skeletal development (Canaza-Cayo et al., 2021). Head measurements (HeL and HeW) were highly correlated (r = 0.93), suggesting proportional cranial development in sheep. Correlations were statistically significant (p < 0.05), confirming the suitability of the data for principal component analysis (Kebede et al., 2022). Most morphometric traits were positively correlated, consistent with coordinated body development; however, a few weak associations indicated relatively independent variation among some traits (Abera et al., 2014). This pattern of positive correlations is typical in livestock populations where overall body size is influenced by coordinated growth processes. Furthermore, the strong correlations observed between body weight and morphometric traits indicate that indirect selection through easily measurable traits could be effective in smallholder systems where weighing scales are unavailable (Deribe et al., 2021). The weakest correlation between shoulder width and rump width (r = 0.06) indicates independent development patterns for these traits, consistent with the findings of Abera et al. (2014), who reported similar independent variation patterns in Ethiopian sheep.

**Table 3:** Phenotypic correlations and their statistical significance levels among body-weight and morphometric traits of ewes.

Trait	BW	HW	CG	BL	CD	RW	RH	RL	PW	SW	CBL	CBC	HeL	HeW	EL	TL	TC
BW	1.00																
HW	**0.87**	1.00															
CG	**0.94**	**0.94**	**1.00**														
BL	**0.90**	**0.88**	**0.93**	**1.00**													
CD	**0.72**	**0.50**	**0.62**	**0.52**	**1.00**												
**RW**	**0.21**	**0.16**	**0.17**	**0.14**	**0.18**	**1.00**											
RH	**0.92**	**0.91**	**0.92**	**0.86**	**0.65**	**0.19**	**1.00**										
RL	**0.63**	**0.50**	**0.55**	**0.57**	**0.55**	**0.16**	**0.56**	**1.00**									
PW	**0.75**	**0.89**	**0.87**	**0.80**	**0.38**	0.07	**0.80**	**0.34**	**1.00**								
SW	**0.66**	**0.83**	**0.79**	**0.79**	**0.24**	0.06	**0.73**	**0.27**	**0.88**	**1.00**							
CBL	**0.77**	**0.85**	**0.82**	**0.75**	**0.46**	**0.11**	**0.83**	**0.46**	**0.80**	**0.77**	**1.00**						
CBC	**0.76**	**0.88**	**0.85**	**0.86**	**0.34**	**0.10**	**0.78**	**0.35**	**0.88**	**0.92**	**0.79**	**1.00**					
HeL	**0.72**	**0.90**	**0.82**	**0.82**	**0.31**	0.08	**0.79**	**0.34**	**0.90**	**0.90**	**0.81**	**0.93**	**1.00**				
HeW	**0.69**	**0.85**	**0.78**	**0.83**	**0.26**	0.08	**0.74**	**0.31**	**0.85**	**0.91**	**0.77**	**0.93**	**0.93**	**1.00**			
EL	**0.70**	**0.84**	**0.77**	**0.69**	**0.40**	**0.10**	**0.76**	**0.32**	**0.81**	**0.79**	**0.83**	**0.80**	**0.84**	**0.80**	**1.00**		
TL	**0.90**	**0.89**	**0.92**	**0.93**	**0.54**	**0.14**	**0.88**	**0.61**	**0.77**	**0.77**	**0.76**	**0.85**	**0.80**	**0.79**	**0.72**	**1.00**	
TC	**0.70**	**0.86**	**0.81**	**0.74**	**0.33**	**0.10**	**0.76**	**0.28**	**0.88**	**0.88**	**0.78**	**0.89**	**0.90**	**0.87**	**0.83**	**0.76**	**1.00**

Within each column, values with different superscript letters differ significantly (Holm-adjusted Wilcoxon signed-rank tests, P < 0.05); values sharing a letter do not differ significantly; RMSE = Root Mean Square Error; MAE = Mean Absolute Error; SD = Standard Deviation; CG= chest girth; MLR= multiple linear regression; PC= principal component.

Correlation coefficients in bold are significant (p < 0.05) (two-tailed); coefficients not in bold are non-significant (p > 0.05) for all correlation coefficients; Bodyweight (BW) and morphometric traits namely height-at-withers (HW), body-length (BL), chest-depth (CD), chest-girth (CG),rump-width (RW), rump-length (RL), rump-height (RH), pelvic-width (PW), shoulder- width (SW), head-width (HeW), head-length (HeL), cannon-bone-length (CBL), cannon-bone- circumference (CBC), ear-length (EL), tail-length (TL), and tail-circumference (TC)

### 3.3 Principal component analysis

In the present study, the application of Principal Component Analysis (PCA) to the 16 morphometric traits of Hararghe Highland ewes was statistically validated. The Kaiser-Meyer-Olkin (KMO) measure of sampling adequacy was exceptionally high (0.93), and Bartlett’s test of sphericity was highly significant (p < 0.001). These metrics confirmed that the partial correlations among the variables were low and that true underlying factors existed within the dataset, satisfying the criteria for factor analysis (Eyduran et al., 2010). This KMO value exceeds those reported in similar morphometric evaluations of Ethiopian sheep (0.91; Kebede & Asaminew, 2023) and West African Dwarf sheep (0.886; De Campos et al., 2014), highlighting the robustness of the sampling and strong inter-correlations among body measurements in this sheep population.

#### 3.3.1 Eigenvalues, percentage of total variance explained, rotated component matrix, and communalities

The eigenvalues, varimax-rotated component loadings, and communality estimates for the 16 morphometric traits are presented in Table 4. This table summarizes the proportion of total variance accounted for by each of the retained principal components following orthogonal rotation of the component matrix. Eigenvalues reflect each principal component's contribution to explaining total variance, with the first component capturing the largest share. Varimax rotation simplifies the factor structure by redistributing loadings so that each variable loads more strongly on a single component. Communalities (ranging 0–1) indicate the proportion of each variable's variance explained by the retained components; higher values suggest adequate representation, while lower values indicate substantial unexplained variance.

**Table 4:** Eigenvalues and shares of total variance along with factor loadings of ewes.

Trait	PC1	PC2	Communalities
Height at Withers	**0.88161**	**0.39804**	0.93568
Chest Girth	**0.81130**	**0.52198**	0.93068
Body Length	**0.79795**	**0.47684**	0.86410
Chest Depth	0.21878	**0.82245**	0.72430
Rump Width	−0.01151	**0.41670**	0.17377
Rump Height	**0.75606**	**0.57113**	0.89781
Rump Length	0.21939	**0.80004**	0.68819
Pelvic Width	**0.91958**	0.18767	0.88085
Shoulder Width	**0.95102**	0.04778	0.90671
Cannon Bone Length	**0.81366**	0.34512	0.78114
Cannon Bone Circumference	**0.94786**	0.16482	0.92559
Head Length	**0.95459**	0.13494	0.92945
Head Width	**0.94807**	0.08699	0.90641
Ear Length	**0.85819**	0.19791	0.77566
Tail Length	**0.78338**	**0.51581**	0.87974
Tail Circumference	**0.92910**	0.11509	0.87647
Eigenvalue	11.50	1.57	
% of total variance	71.89	9.84	81.73

Principal component analysis reduced the 16 inter-correlated morphometric traits to two orthogonal principal components (PCs) with eigenvalues greater than 1 (11.50 and 1.57, respectively). These two components jointly explained 81.73% of the total phenotypic variance. This level of cumulative variance is consistent with previous reports; Kebede and Asaminew (2023) found that the first two principal components accounted for 82.4% of the variance in indigenous Ethiopian sheep, while De Campos et al. (2014) reported 85.08% in West African Dwarf sheep. In contrast, a lower cumulative variance (67.8%) was reported by Yadav et al. (2016) in the Madgyal sheep population. This high proportion of explained variance indicates effective data reduction and suggests that most of the underlying morphological variation can be captured by a limited number of independent components.

A high proportion of this demonstrates effective data reduction. The scree-parallel analysis plot (Fig. 2) further validated the retention of two components. The pronounced "elbow" inflection point after the second component demonstrated that subsequent components contributed negligible explanatory power relative to random variation, confirming the appropriateness of the Kaiser-Guttman criterion for this dataset (Canaza-Cayo et al., 2021).

**Fig. 2:**
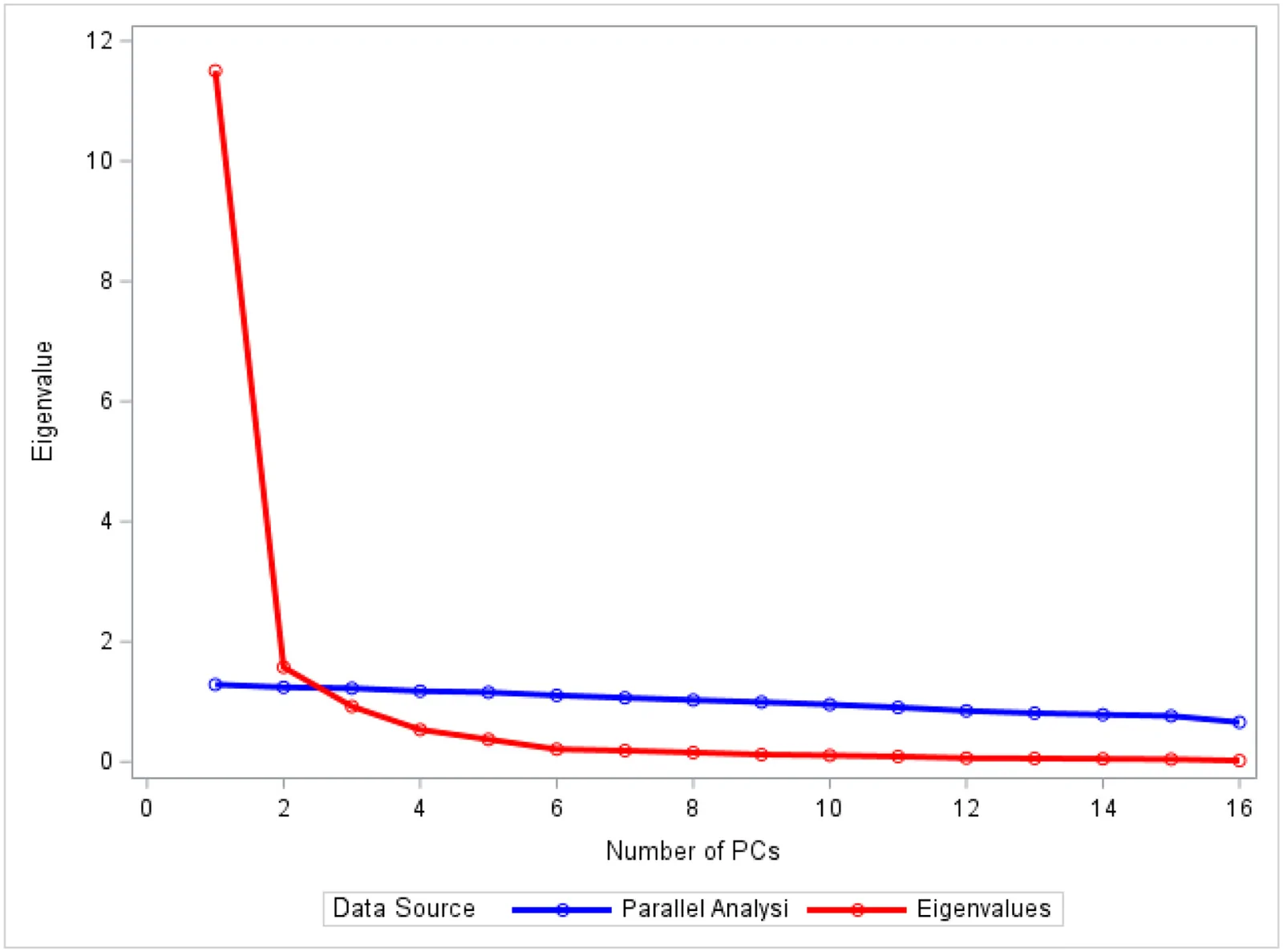
Scree and parallel analysis plots for principal component extraction from 16 morphometric traits of Hararghe Highland ewes (n = 392). The intersection of the two lines (after the second component) confirms the retention of two principal components, consistent with the Kaiser-Guttman criterion (eigenvalues > 1.0).

Consistent with multivariate morphometric literature across diverse livestock species, the first principal component (PC1) represented a generalized body size factor. PC1 alone explained 71.89% of the total variance and loaded heavily on overall skeletal and structural dimensions, including head length (0.955), shoulder width (0.951), head width (0.948), cannon bone circumference (0.948), pelvic width (0.920), tail circumference (0.929), and wither height (0.882). This aligns with findings in camels (Rotimi et al., 2024) and various sheep breeds (Mekonnen et al., 2025; Salako, 2006), where PC1 consistently captures the maximum variation related to the overall size and conformation of the animal.

The second principal component (PC2), which explained 9.84% of the variance, acted as a body shape or structural refinement factor. PC2 loaded heavily on traits such as chest depth (0.822), rump length (0.800), rump height (0.571), and rump width (0.417). This orthogonal separation of size and shape is a fundamental advantage of PCA. As noted by Yakubu (2009), while PC1 reflects allometric size, subsequent components lack correlation to overall size and provide taxonomically informative details regarding body proportions, such as thoracic depth and pelvic structure, which are crucial for evaluating breed functionality and meat/milk production suitability (Mekonnen et al., 2025). The communalities extracted in this study ranged widely from 0.174 (RW) to 0.936 (HW). While most traits exhibited high communalities (>0.68), indicating that the extracted factors successfully explained a large proportion of their variance. The low communality observed for rump width (0.174) warrants further investigation. The low communality of rump width indicates that this trait was poorly represented by the two retained components and may reflect a dimension of phenotypic variation that is relatively independent of the dominant body-size and body-shape axes. Alternatively, measurement of rump width between the two tuber coxae may be subject to greater error due to variation in fat deposition or muscle condition. Retaining RW in the analysis ensures comprehensive phenotypic characterization, but its low shared variance suggests it is heavily influenced by environmental factors or field measurement error (Khargharia et al., 2015; Okpeku et al., 2011). For breeding programs, this implies that rump width may be less reliable than other traits for indirect selection.

#### 3.3.2 Scree plot and parallel analysis

Fig. 2 shows the scree plot together with the parallel analysis, and both agree on the same cut-off. Eigenvalues drop sharply after the second component, producing a clear elbow; components beyond this point add little more than random variation, which supports retaining two components under the Kaiser-Guttman rule (Canaza-Cayo et al., 2021). Together with the 81.73% cumulative variance already explained, this indicates that two components capture the bulk of the useful information without overfitting the model. Because PC scores are by construction uncorrelated, extracting them from the original, highly correlated measurements (r = 0.06–0.94) removes the multicollinearity that affected the raw-trait model and gives more stable, interpretable regression coefficients (Tahtali et al., 2019). Reducing 16 correlated traits to two independent factors therefore simplifies the analysis while still reflecting overall body conformation, supporting its use in developing prediction equations relevant to breeding decisions in Hararghe Highland ewes.

### 3.4 Multiple linear regression models for body weight prediction

The regression coefficients, standard errors, t-statistics, significance levels (p-values), variance inflation factors (VIF), and adjusted coefficients of determination (R²) derived from the multiple linear regression analysis are summarized in Table 5.

**Table 5:** MLR of body weight on original morphometric traits and their PC factor scores of ewes.

Model	Coefficient	Standard Error	t-Value	P-value	Tolerance	VIF
Original morphometric traits as predictors						
**Intercept**	−32.47	2.47	−13.17	< .0001	.	.
HW	0.05	0.07	0.74	0.4589	0.05	19.05
CG	0.33	0.05	6.35	< .0001	0.04	28.24
BL	0.32	0.05	6.38	< .0001	0.06	16.11
CD	0.12	0.02	6.11	< .0001	0.36	2.79
RW	0.05	0.02	2.24	0.0260	0.91	1.10
RH	0.25	0.04	5.98	< .0001	0.10	10.01
RL	0.04	0.02	1.73	0.0842	0.45	2.24
PW	−0.07	0.09	−0.79	0.4323	0.10	10.15
SW	−0.52	0.10	−5.47	< .0001	0.11	9.26
CBL	0.07	0.06	1.20	0.2323	0.20	5.08
CBC	0.41	0.13	3.07	0.0023	0.05	18.93
HeL	−0.18	0.12	−1.54	0.1255	0.07	14.85
HeW	−0.28	0.12	−2.46	0.0142	0.06	16.65
EL	0.14	0.07	2.14	0.0334	0.19	5.16
TL	0.08	0.07	1.15	0.2521	0.08	13.08
TC	0.03	0.05	0.68	0.4984	0.12	8.02
	R^2^ adjusted = 0.95					
Orthogonal morphometric traits as predictors						
Intercept	25.67	0.07	387.96	<.0001	1.0	1.0
PC1	2.98	0.07	45.00	<.0001	1.0	1.0
PC2	2.90	0.07	43.76	<.0001	1.0	1.0
		R² adjusted = 0.91				

Within each column, values with different superscript letters differ significantly (Holm-adjusted Wilcoxon signed-rank tests, P < 0.05); values sharing a letter do not differ significantly; RMSE = Root Mean Square Error; MAE = Mean Absolute Error; SD = Standard Deviation; CG= chest girth; MLR= multiple linear regression; PC= principal component.

The present study demonstrated that both multiple linear regression (MLR) models, based on original morphometric traits and principal component (PC) scores, can effectively predict body weight in Hararghe Highland ewes. However, clear differences were observed in terms of statistical robustness, interpretability, and suitability for practical application. To increase meat yield and optimize sheep production, the genetic improvement of body weight is a primary objective, which requires a thorough understanding of correlated morphometric traits. The multiple linear regression (MLR) analysis using the original 16 morphometric traits yielded a highly predictive model (adjusted R² =0.95), with the traits such as chest girth, body length, chest depth, rump height, rump width, shoulder width, cannon bone circumference, head width, and ear length were significant predictors. The higher apparent predictive accuracy obtained using original morphometric traits (adjusted R² = 0.95) is consistent with previous studies that reported strong relationships between body weight and linear body measurements in sheep. For instance, Kebede and Asaminew (2023) reported an R² value of 0.97 when predicting body weight using multiple morphometric traits in Ethiopian sheep populations. Similarly, Khan et al. (2014) demonstrated high predictive accuracy using multiple regression models in Harnai sheep. These findings confirm that incorporating several body measurements can enhance predictive performance. All 16 traits were purposely retained in this model, rather than reduced via stepwise selection, to explicitly demonstrate the severity of multicollinearity that arises when utilizing multiple highly inter-correlated body measurements simultaneously, thereby justifying the necessity of the PCA approach; several traits with non-significant coefficients (e.g., HW, PW, CBL, and TC) nonetheless show inflated standard errors consistent with the multicollinearity (Table 5).

Multicollinearity is a well-recognized limitation in livestock morphometric analyses (Ibe, 1989; Khan et al., 2014; Yakubu, 2009), particularly when highly correlated original predictors are included in regression models. In the present study, several key traits with strong predictive capacity such as chest girth (VIF = 28.24), body length (VIF = 16.11), and head width (VIF = 16.65), exhibited variance inflation factor values substantially exceeding the critical threshold of 10, indicating severe multicollinearity. Such high levels of collinearity have been reported to produce unstable least-squares estimates and inflated standard errors (Rook et al., 1990), thereby limiting robust inference and hindering the biological interpretation of individual trait effects. To address this limitation, the present study applied principal component analysis to transform correlated variables into orthogonal components. The PC-based regression model successfully eliminated multicollinearity (VIF = 1.0) while maintaining high predictive accuracy (adjusted R² = 0.91). This finding is in strong agreement with Kebede et al. (2021), who demonstrated that regression models based on PC scores provide stable and reliable predictions in Ethiopian local chickens. Eyduran et al. (2010) reported that the use of factor scores in regression analysis improves model stability and eliminates multicollinearity without substantial loss of information. Similarly, De Campos et al. (2014) used principal component scores to predict body weight in West African Dwarf sheep, confirming that orthogonal variables provide a more statistically robust alternative to original inter-correlated traits. Therefore, the application of principal component factor scores in regression models is better for interpretation and composite indices to livestock breed improvement program. While calculating exact PC scores requires statistical software, the practical utility of this model for smallholder farmers and extension workers lies in its identification of the most informative, non-redundant trait clusters. Farmers can focus their selection and marketing decisions on the highly loading traits of PC1 (such as, chest girth, body length, and height at withers) as reliable, easily measurable proxies for overall body weight and conformation, rather than attempting to measure all 16 traits.

Chest girth (CG); height-at-withers (HW); body length (BL); chest depth (CD); rump width (RW); rump length (RL); rump height (RH); pelvic width (PW); shoulder width (SW); head width (HeW); head length (HeL); cannon bone length (CBL); cannon bone circumference (CBC); ear length (EL); tail-length (TL); and tail-circumference (TC).

### 3.5 Model comparison and predictive performance

The cross-validation results showed clear differences in predictive performance among the five models (Friedman test: RMSE, χ² = 32.83, df = 4, P < 0.0001; R², χ² = 32.83, df = 4, P < 0.0001; MAE, χ² = 32.58, df = 4, P < 0.0001; Kendall’s W ≈ 0.82). The full multiple linear regression (MLR) model achieved the highest numerical predictive accuracy (R² = 0.940; RMSE = 1.034 kg; MAE = 0.796 kg), followed closely by LASSO (R² = 0.939; RMSE = 1.047 kg; MAE = 0.815 kg) and Elastic Net (R² = 0.939; RMSE = 1.047 kg; MAE = 0.811 kg). Pairwise comparisons with Holm adjustment showed that MLR, LASSO, and Elastic Net each performed significantly better than PC regression and the chest-girth-only model (Holm-adjusted P = 0.0195 for each of these comparisons). In contrast, the three best-performing models did not differ significantly from one another (adjusted P ≥ 0.58). PC regression outperformed the chest-girth-only model for R² and RMSE (Holm-adjusted P = 0.0195), but the two did not differ for MAE. These results indicate that LASSO and Elastic Net achieved predictive performance comparable to the full MLR model while providing more stable coefficient estimates under strong multicollinearity among the morphometric predictors. The PC regression model showed lower predictive accuracy than the three best-performing models (R² = 0.905; RMSE = 1.307 kg; MAE = 1.027 kg), although it substantially reduced the dimensionality of the predictor set and eliminated multicollinearity. Its lower predictive performance suggests that dimension reduction resulted in some loss of information relevant to body-weight prediction. Nevertheless, PC regression remains useful when the objective is to summarize correlated morphometric traits into a small number of interpretable dimensions rather than to maximize prediction accuracy alone. The chest-girth-only model had the lowest predictive accuracy among the models evaluated (R² = 0.881; RMSE = 1.460 kg; MAE = 1.149 kg), but its simplicity gives it an important practical advantage under field conditions where weighing scales and statistical software are unavailable (Table 6).

**Table 6:** Predictive performance of different regression models evaluated using 10-fold cross-validation.

Model	R² (mean ± SD)	RMSE, kg (mean ± SD)	MAE, kg (mean ± SD)
Single trait (CG)	0.881 ± 0.032^a^	1.460 ± 0.174^a^	1.149 ± 0.142^a^
MLR (16 traits)	0.940 ± 0.019^c^	1.034 ± 0.156^c^	0.796 ± 0.105^b^
PC regression	0.905 ± 0.025^b^	1.307 ± 0.164^b^	1.027 ± 0.135^a^
LASSO	0.939 ± 0.018^c^	1.047 ± 0.150^c^	0.815 ± 0.103^b^
Elastic Net	0.939 ± 0.019^c^	1.047 ± 0.155^c^	0.811 ± 0.108^b^

Within each column, values with different superscript letters differ significantly (Holm-adjusted Wilcoxon signed-rank tests, P < 0.05); values sharing a letter do not differ significantly; RMSE = Root Mean Square Error; MAE = Mean Absolute Error; SD = Standard Deviation; CG= chest girth; MLR= multiple linear regression; PC= principal component.

## 4 Conclusion

Principal component analysis reduced the 16 correlated morphometric traits of Hararghe Highland ewes to two orthogonal components representing overall body size and body shape, which together explained 81.73% of the total variance. Regression on PC scores eliminated multicollinearity (VIF = 1.0), compared with severe multicollinearity in the original-trait MLR model (VIF > 10 for nine of 16 traits), but resulted in lower predictive accuracy. In 10-fold cross-validation, MLR achieved the highest numerical accuracy (R² = 0.940; RMSE = 1.034 kg), while LASSO and Elastic Net produced comparable performance (R² = 0.939; RMSE = 1.047 kg) and significantly outperformed PC regression and the chest-girth-only model (Holm-adjusted P < 0.05). Thus, LASSO and Elastic Net provide attractive alternatives to full MLR when prediction accuracy and multicollinearity control are both important. PC regression, although less accurate for prediction, provides a parsimonious and interpretable representation of body conformation. Because each PC score is calculated from all 16 measurements, PCA reduces the number of variables analysed but not the number of measurements required. For practical field estimation where statistical software and weighing scales are unavailable, chest girth alone provides a simple option, although its prediction error was higher (RMSE = 1.460 kg). These findings are specific to Hararghe Highland ewes under the conditions represented in this study. Further validation across age classes, sexes, seasons, management systems, and independent populations is needed before broader application. Genetic studies are also warranted to determine whether the phenotypic variation represented by the PC scores has a heritable basis.

## Ethics statement

Ethical approval for this study was obtained from the School of Animal and Range Sciences Review Approval Committee, Haramaya University, Ethiopia (Approval ID: SARS 051/24). Verbal informed consent was obtained from all participating livestock owners prior to the inclusion of their animals in this research.

## Generative AI declaration

No generative AI or AI-assisted technologies were used in the preparation of this manuscript.

## Ethics declaration

This study was conducted in accordance with the ARRIVE (Animal Research: Reporting of In Vivo Experiments) guidelines. This study was approved by the School of Animal and Range Sciences Review Approval Committee, Haramaya University, Ethiopia. (Approval No. Approval ID: SARS 051/24)

## Funding

This research did not receive any specific grant from funding agencies in the public, commercial, or not-for-profit sectors. Dambi Dollo University provided salary support to the corresponding author, and Haramaya University provided research facilities and field-travel logistics.

## CRediT authorship contribution statement

**Mohammed Yousuf:** Writing – original draft, Methodology, Formal analysis, Data curation, Conceptualization. **Kefelegn Kebede:** Supervision, Software, Methodology. **Yesihak Yusuf:** Writing – review & editing, Supervision. **Gemeda Duguma:** Writing – review & editing, Formal analysis. **Solomon Abegaz:** Writing – review & editing, Validation.

## Declaration of competing interest

The authors declare that they have no known competing financial interests or personal relationships that could have appeared to influence the work reported in this paper.

## Data Availability

Data reported in this study are available upon request to the authors.
